# Primary Central Nervous System Vasculitis as an Unusual Cause of Intracerebral Hemorrhage: A Case Report

**DOI:** 10.7759/cureus.13847

**Published:** 2021-03-12

**Authors:** Mariem Borcheni, Basel Abdelazeem, Bilal Malik, Simhachalam Gurugubelli, Arvind Kunadi

**Affiliations:** 1 Internal Medicine, University of Sfax Faculty of Medicine, Sfax, TUN; 2 Internal Medicine, McLaren Health Care, Flint/Michigan State University, Flint, USA; 3 Internal Medicine/Nephrology, McLaren Health Care, Flint/Michigan State University, Flint, USA

**Keywords:** primary cns vasculitis, cns angiitis, intracerebral hemorrhage, induction therapy

## Abstract

A 64-year-old male with a history of transverse myelitis presented to the hospital with a decreased level of consciousness of one day's duration. CT of the head revealed intracranial hemorrhage measuring 2 x 1.2 cm in the right temporal lobe and multiple small hemorrhages in the left hemisphere, suggestive of vasculitis. Initial vasculitis workup was negative for antinuclear antibody (ANA), complement component 3 (C3), and antineutrophil cytoplasmic antibodies: P-ANCA, C-ANCA. Syphilis, hepatitis B and C, West Nile virus antibody [immunoglobulin G (IgG) and immunoglobulin M (IgM)], herpes simplex virus (HSV) polymerase chain reaction (PCR), and HIV 1 and 2 were also negative. In view of the CT scan findings suggestive of vasculitis and the vague presentation of primary central nervous system vasculitis (PCNSV), a brain biopsy was performed. It revealed angiocentric granulomatous inflammation with focal vessel disruption and associated parenchymal hemorrhage, consistent with a diagnosis of granulomatous vasculitis. The patient received levetiracetam, multiple high doses of steroids, and six cycles of cyclophosphamide for a six-month duration. After induction, he has remained in remission without any maintenance therapy until now (eight years post-presentation).

## Introduction

Primary central nervous system vasculitis (PCNSV) is an inflammatory disease affecting the blood vessels of the brain and spinal cord in the absence of an underlying systemic inflammatory disease. It has a predilection for leptomeningeal, subcortical, and cortical arteries. PCNSV is considered a diagnosis of exclusion since it is a rare disease with an annual incidence rate of 2.4 cases per one million inhabitants in the United States [1]. Despite its rarity, a delayed diagnosis of the condition can lead to devastating outcomes in the patient. PCNSV has been reported to occur in all ages, with a median age of diagnosis of 50. There is no significant difference in incidence between genders (i.e., a specific gender is not predisposed to developing PCNSV) [1]. Primary angiitis can cause stroke in younger patients without cerebrovascular risk factors, with approximately 3-5% of strokes in patients aged <50 years occurring secondary to PCNSV [2].

The pathogenesis of PCNSV remains unclear; however, it is thought to be an autoimmune response secondary to viral and bacterial agents targeting both small and large vessels of the CNS [3]. Viral agents may include the varicella-zoster virus, Cytomegalovirus, Epstein-Barr virus, HIV, and hepatitis C virus. Bacterial agents may include Mycoplasma, Rickettsia, and Treponema. Connective tissue disorders and systemic vasculitides like systemic lupus erythematosus, Churg-Strauss syndrome, Behcet's syndrome, and Sjögren's syndrome have also been implicated [1,3]. Cerebral amyloid angiopathy is another potential trigger [3].

The autoimmune response is based specifically on T cells, which infiltrate CNS blood vessels and cause thickening of the wall with alternating segmental stenosis. In a single case report, extensive CD45R0+ T cell infiltration was noted around small CNS arteries of a biopsy sample using immunohistochemical staining [4]. It has also been demonstrated that matrix metalloproteinases, particularly MMP-9, are one of the major effector molecules in animal models of PCNSV [4]. The inflammatory process may also cause weakening and destruction of the vessel wall, thereby leading to blood vessel rupture and progression to intracranial hemorrhage [5].

PCNSV has a wide range of non-specific clinical manifestations ranging from headache and cognitive dysfunction to stroke. Intracerebral hemorrhage (ICH) is one of its rarest presenting features, occurring in a mere 12% of cases [2].

Remission of PCNSV can be achieved with induction therapy with or without maintenance therapy. However, relapses were encountered in 50% of patients who received only induction therapy with a median follow-up of 57 months [6]. In this report, we present a case of a biopsy-proven PCNSV presenting with ICH; the patient has remained in remission for eight years after receiving induction therapy only.

## Case presentation

Our patient was a 64-year-old male with a past medical history remarkable for multiple urinary tract infections, urosepsis, and transverse myelitis diagnosed one year prior to presentation, as well as deep vein thrombosis treated with anticoagulation. He presented with a decreased level of consciousness of one day's duration and personality changes (low anger threshold and mood changes) for three weeks. The patient had never had these episodes before. He had a blood pressure of 130/70 mmHg, heart rate of 89 beats per minute, temperature of 36.7 °C, respiratory rate of 18 breaths per minute, and oxygen saturation of 97% on room air. Of note, the patient had an altered baseline cognitive function and required assistance for performing his daily activities.

**Figure 1:**
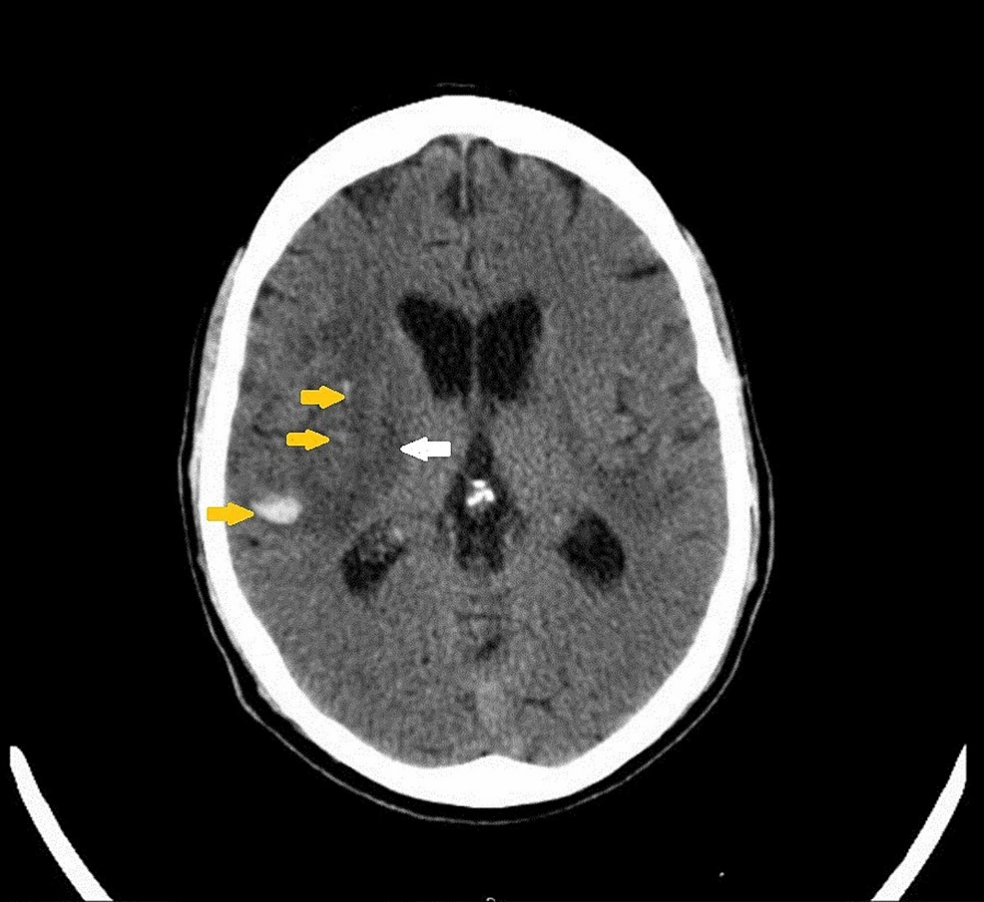
CT scan of the head without contrast The image demonstrates small amounts of acute intraparenchymal hemorrhage in the right temporal and frontal lobes (yellow arrows) with a large area of associated edema below (white arrow) CT: computed tomography

On examination, he was comatose with a Glasgow coma scale score of 6. There was significant cogwheel rigidity of the left upper extremity with a near-normal tone on the right side. Bilateral atrophy of the lower extremities, more pronounced on the left, was noted with bilateral foot drop and positive Babinski sign, bilaterally. Blood cell counts, hepatic enzymes, ferritin, blood urea nitrogen (BUN), urine drug screen, and creatinine levels were all within normal limits. Urine and blood cultures were drawn and sent but returned negative. CT scan of the head was performed and demonstrated an intracranial hemorrhage measuring 2 x 1.2 cm, with 6.5 mm extension craniocaudally, in the right temporal lobe. Figure 1 demonstrates the large area of associated edema.

**Figure 2:**
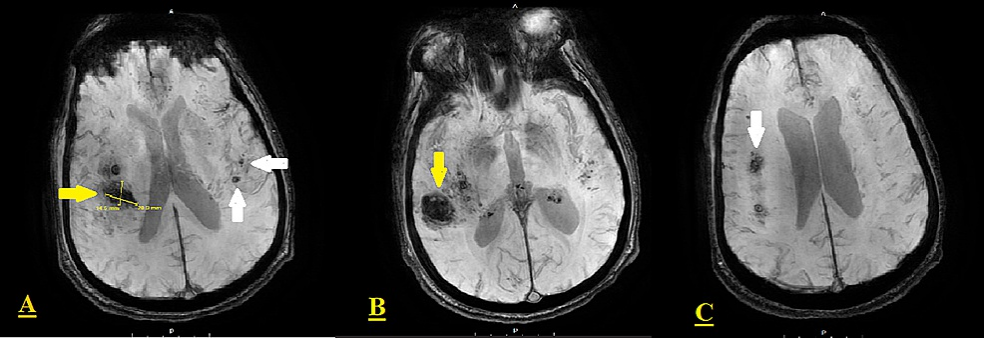
Brain MRI (T2*-weighted gradient-echo sequences) The images reveal focal intraparenchymal hemorrhage with surrounding edema in the right posterior, superior temporal lobe (yellow arrows in A-B). Additional areas of hemorrhage are present superiorly in the right frontal lobe (white arrow in C), and the left hemisphere below (white arrows in A) MRI: magnetic resonance imaging

These findings were confirmed by an MRI of the brain, which also revealed an additional area of hemorrhage in the right frontal lobe, with a large amount of surrounding edema but no midline shift, and smaller hemorrhages in the left hemisphere suggesting vasculitis (Figures 2A-2C).

Lumbar puncture was performed and repeated three weeks later. Their results are summarized in Table 1.

**Table 1:** CSF analysis CSF: cerebrospinal fluid; LP: lumbar puncture; RBC: red blood cells; WBC: white blood cells; PMN: polymorphonuclear leukocyte; EOS: eosinophils

Test name		LP1	LP2	Reference range
CSF cell count	CSF tube number	4	4	
	CSF volume (ml)	10.0	8.0	
	CSF appearance	Clear	Clear (no difference in color was observed)	
	CSF XANTH	Marked	Present	
	Total RBC (/cmm)	11	0	0-5
	Total WBC (/cmm)	84	78	0-7
	PMN (%)	2	1	
	Lymphs (%)	91	96	
	Monos (%)	6	2	
	EOS (%)	1	1	
CSF glucose (mg/dl)		32	68	50-80
CSF total protein (mg/dl)		171	120	12-60
Culture, CSF (includes Gram stain)		No growth on final reading		
Oligoclonal bands		Negative		
VDRL (CSF)		Non-reactive		
West Nile virus antibody, IgG Ab CS		<1.3		<1.30: negative; 1.30-1.49: equivocal; >1.49: positive
West Nile Virus antibody, IgM Ab CS		<0.90		<0.90: negative; 0.90-1.10: equivocal; >1.10: positive

Initial vasculitis workup was negative for antinuclear antibody (ANA), complement component 3 (C3), and antineutrophil cytoplasmic antibodies: P-ANCA, C-ANCA. Syphilis, hepatitis B and C, West Nile virus antibody [immunoglobulin G (IgG) and immunoglobulin M (IgM)], herpes simplex virus (HSV) polymerase chain reaction (PCR), HIV 1 and 2, and hypogammaglobulinemia with serum immunofixation levels were also negative.

**Figure 3:**
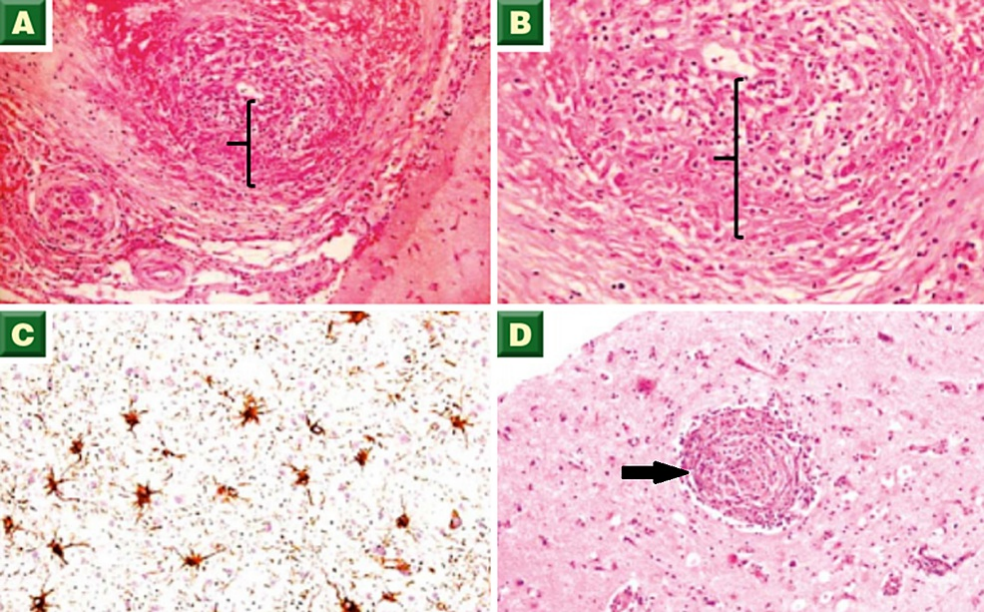
Biopsy images - 1 (A, B) Transmural granulomatous inflammation of leptomeningeal arteries composed predominantly of lymphocytes and scattered histiocytes (brackets, H&E stain). (C) Vessel wall with membranous staining (immunohistochemistry staining). (D) Cerebral parenchyma showing a focal vessel with concentric perivascular inflammation consisting of chronic inflammatory cells (arrow, H&E stain) H&E: hematoxylin and eosin

**Figure 4:**
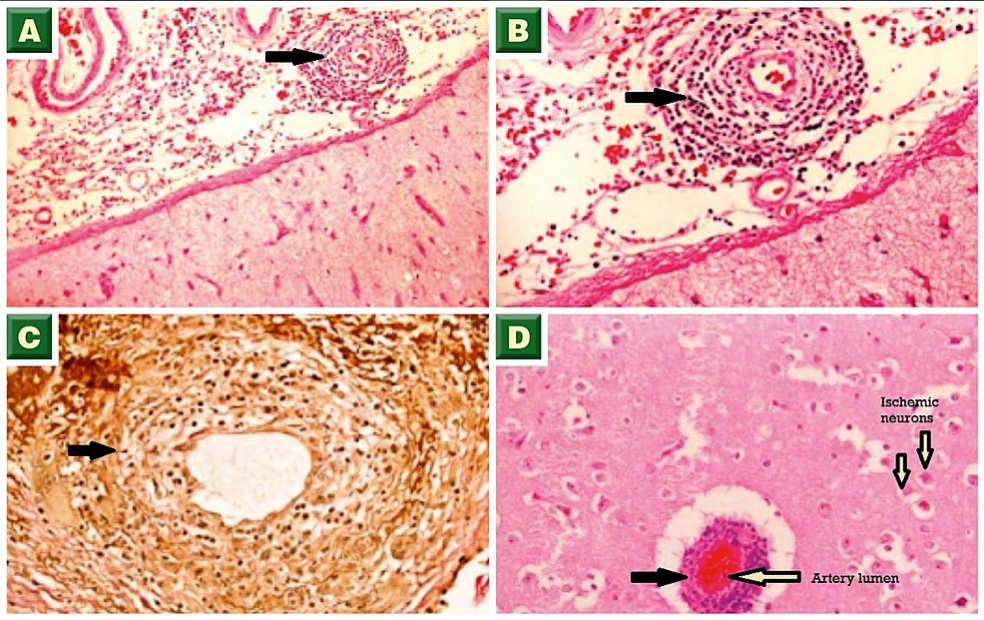
Biopsy images - 2 (A, B) Cerebral parenchymal surface showing vessels with concentric perivascular chronic inflammatory cells (arrows, H&E stain). (C) Vessel wall inflammation with diffuse staining (arrow, immunohistochemistry staining). (D) Cerebral parenchyma showing a congested vessel with perivascular inflammation (black arrow) and adjacent red neurons (H&E stain) H&E: hematoxylin and eosin

In view of CT and MRI findings suggestive of vasculitis, and a vague initial presentation, a brain biopsy was performed for further workup. The biopsy revealed angiocentric granulomatous inflammation with focal vessel disruption and associated parenchymal hemorrhage, consistent with a diagnosis of granulomatous vasculitis (Figures 3A-3D, 4A-4D).

The anticoagulation was discontinued and an inferior vena cava filter (IVC) filter was placed. The patient received levetiracetam 1,000 mg/day, multiple high doses of steroids, and six cycles of cyclophosphamide, each administered one month apart. The dosage of cyclophosphamide was 870 mg/m^2^ per cycle in the first three cycles followed by 945 mg/m^2^ for the subsequent cycles.

The patient was followed up every four to six weeks during the first two years, and subsequently every four months until now. After induction, he has remained in remission without any maintenance therapy to date (eight years post-presentation).

## Discussion

Clinical features

Clinical presentations of this condition are highly variable and non-specific. These may range from acute to progressive, chronic, and even relapsing/remitting. The absence of clinical manifestations is also very common in CNS vasculitis [7], with the most frequent presentations being headache (60% of cases) and altered cognition (50% of cases). Encephalopathy, seizures, hemiparesis, transient ischemic attack, stroke, dysarthria, aphasia, and ataxia have also been documented [8]. Constitutional symptoms like fever, weight loss, and night sweats are infrequent and rather suggestive of systemic vasculitis. Our patient presented with personality changes and decreased level of consciousness associated with focal neurological deficits in the absence of constitutional symptoms.

Of note, 5% of primary CNS vasculitis affects the spinal cord. However, spinal cord vasculitis being the only manifestation of PCNSV is rare, and patients with spinal involvement have concomitant or subsequent intracranial involvement during their disease course [9]. To facilitate the initial recognition of PCNSV, four clinical presentations orienting to primary CNS angiitis have been described [10]: 

1) Recurrent cerebral ischemia and/or ischemia affecting multiple vascular beds with the presence of inflammatory changes in the cerebrospinal fluid (CSF)

2) Chronic meningitis in the absence of infectious or malignant etiology

3) Cerebral ischemia in young patients without cerebrovascular risk factors for strokes

4) Subacute/chronic headache in the setting of cognitive dysfunction and/or chronic aseptic meningitis.

Differential diagnosis

The differential diagnosis of PCNSV is very broad, making the diagnosis challenging. Most of the tests performed in patients with suspected PCNSV are focused on ruling out mimics. The main PCNSV mimic is reversible cerebral vasoconstriction syndrome (RCV), which occurs mainly in the postpartum period and after exposure to vasoconstrictive medications (triptans, amphetamines, selective serotonin reuptake inhibitors, cocaine) [11]. Infectious arteritis is an important mimic to rule out since, in this setting, immunosuppressive treatment can lead to disastrous outcomes. The varicella-zoster virus infection is an important entity to consider because it is also associated with the primary CNS vasculitis, usually when signs of viral infection are absent [12]. Other mimics that should be considered are intracranial atherosclerosis, cerebral embolism, primary intravascular CNS lymphoma, multiple sclerosis, and gliomatosis cerebri. Secondary CNS vasculitis is usually associated with features suggesting an underlying systemic disease like granulomatosis with polyangiitis, systemic lupus erythematosus, and Behcet’s disease among others.

Diagnosis

Diagnostic criteria were first proposed in 1988 to distinguish between PCNSV and mimics [13]. These criteria are based on the presence of unexplained neurologic deficits after exhaustive evaluation, and the existence of angiographic or pathologic characteristics of CNS angiitis with no proof of any disease that could mimic PCNSV.

Diagnosis of PCNSV is based on complete patient history and physical examination looking for symptoms and signs orienting to secondary CNS vasculitis or PCNSV mimics.

Laboratory workup can help orient the diagnosis, but there is no test to confirm or rule out PCNSV. Although acute-phase reactants (C-reactive protein and erythrocyte sedimentation rate) and serologic markers like antinuclear antibodies (ANA) and ANCA are usually normal in PCNSV, it is still mandatory to measure them in order to rule out systemic disease. Blood cultures and serologies should be done to evaluate common infectious etiologies.

CSF analysis is a crucial test and it has to be done for all PCNSV-suspected patients in the absence of contraindications. This test has a high negative predictive value, making it a valuable tool for ruling out CNS vasculitis. CSF results are, however, non-specific in 80-90% of PCNSV cases. They commonly demonstrate lymphocytic pleocytosis, elevated CSF protein, and normal glucose levels. Our patient was found to have the aforementioned CSF analysis results. Occasionally, high CSF IgG to serum IgG ratio and oligoclonal bands are present on analysis as well [10]. 

MRI is the imaging method of choice for patients with suspected CNS vasculitis due to its high sensitivity (95%). Thus, a normal MRI brain, especially in the context of normal CSF analysis, makes PCNSV very improbable. A recent study demonstrated that bilateral, small/medium vessel involvement, multi-territorial vascular occlusions, and leptomeningeal enhancement are common findings in patients with PCNSV [14]. Parenchymal hemorrhage and tumor-like lesions were reported on imaging in 8-55% and 5-10% of cases, respectively [14-16]. In our patient, the MRI was significant for bilateral intracranial hemorrhages with the largest measuring 2 x 1.2 cm. 

Brain biopsy remains the gold standard diagnostic tool of CNS vasculitis despite its low sensitivity and false-negative rate of 25% [7]. It is a powerful method to confirm vasculitis and to rule out mimics, particularly infectious and malignant etiologies. The diagnosis of PCNSV is confirmed by the presence of transmural lymphocytic infiltrate and vascular wall damage (with or without transmural fibrinoid necrosis) on biopsy [5]. Three histopathologic patterns have been identified: granulomatous, lymphocytic, and necrotizing. In a study conducted on patients with PCNSV, surgical biopsies showed that 56% of cases have granulomatous patterns (27% were associated with β-A4 amyloid deposition). Lymphocytic pattern and acute necrotizing pattern were seen in 20% and 22% of cases, respectively [5]. For our patient, the biopsy revealed granulomatous vasculitis associated with parenchymal hemorrhage.

Based on clinical, imaging, and pathological characteristics, six subtypes of PCNSV have also been identified: angiogram-negative biopsy-positive, amyloid-β-related cerebral angiitis, prominent meningeal enhancement in MRI, spinal cord involvement, rapidly progressive PCNSV, and PCNSV presenting with intracranial or subarachnoid hemorrhage - which was the one found in our patient. 

Treatment

PCNSV can be successfully treated with immunosuppressive therapy. After confirming the diagnosis, corticosteroids plus cyclophosphamide is the commonly used initial therapy with a goal of remission induction. Alternative diagnoses should be considered if no response to corticosteroids and cyclophosphamide is noted prior to attempting alternative therapies. Rituximab can be used as an alternative to cyclophosphamide for induction therapy [17]. Many medications in PCNSV regimens are significantly toxic, necessitating patient education and monitoring for side effects. Corticosteroid use can be associated with Cushingoid appearance, hypertension, diabetes mellitus, osteoporosis, and opportunistic infections. Urothelial toxicity, infection, and infertility are the main side effects of cyclophosphamide. 

Patients should be evaluated every four to six weeks for clinical response and the development of adverse drug reactions/side effects. Complete blood count, liver function test, urinalysis, and neuroimaging may be appropriate considerations during these interval follow-ups. After achieving clinical response, the decision to taper corticosteroids can be made. Three to six months of cyclophosphamide is generally sufficient for the majority of patients to achieve remission. At remission, consideration should be made to switch to less toxic medication for an additional 6-12 months of maintenance therapy. Azathioprine and mycophenolate mofetil are options for maintenance therapy [18]. Our patient did not receive maintenance therapy due to total remission after induction treatment with corticosteroids and cyclophosphamide for a duration of six months. 

## Conclusions

This case highlights intracranial hemorrhage as a rare manifestation of PCNSV in a 64-year-old male. Patients diagnosed with PCNSV usually receive induction therapy to achieve remission, followed by maintenance therapy to maintain it. The patient presented in this article has remained in remission for eight years after receiving only induction therapy.
